# Blood Culture Result Profile in Patients With Central Line-Associated Bloodstream Infection (CLABSI): A Single-Center Experience

**DOI:** 10.7759/cureus.40202

**Published:** 2023-06-09

**Authors:** Tetsuya Akaishi, Koichi Tokuda, Makoto Katsumi, Shin-ichi Fujimaki, Tetsuji Aoyagi, Hideo Harigae, Tadashi Ishii

**Affiliations:** 1 Regional Medicine, Tohoku University Hospital, Sendai, JPN; 2 Infection Control, Tohoku University, Sendai, JPN; 3 Laboratory Medicine, Tohoku University Hospital, Sendai, JPN; 4 Microbiology and Infectious Diseases, Toho University, Tokyo, JPN; 5 Hematology, Tohoku University, Sendai, JPN; 6 Kampo and Integrative Medicine, Tohoku University Hospital, Sendai, JPN

**Keywords:** blood stream infections, staphylococcus epidermidis bacteremia, central venous access device, central line associated bloodstream infection, blood cultures

## Abstract

Background

Central line-associated bloodstream infection (CLABSI) is among the most common bloodstream infections in the university hospital and intensive care unit settings. This study evaluated the routine blood test findings and microbe profiles of bloodstream infection (BSI) by the presence and types of central vein (CV) access devices (CVADs).

Methods

A total of 878 inpatients at a university hospital who were clinically suspected for BSI and underwent blood culture (BC) testing between April 2020 and September 2020 were enrolled. Data regarding age at BC testing, sex, WBC count, serum C-reactive protein (CRP) level, BC test results, yielded microbes, and usage and types of CVADs were evaluated.

Results

The BC yields were detected in 173 patients (20%), suspected contaminating pathogens in 57 (6.5%), and 648 (74%) with a negative yield. The WBC count (p=0.0882) and CRP level (p=0.2753) did not significantly differ between the 173 patients with BSI and the 648 patients with negative BC yields. Among the 173 patients with BSI, 74 used CVADs and met the diagnosis of CLABSI; 48 had a CV catheter, 16 had CV access ports, and 10 had a peripherally inserted central catheter (PICC). Patients with CLABSI showed lower WBC counts (p=0.0082) and serum CRP levels (p=0.0024) compared to those with BSI who did not use CVADs. The most commonly yielded microbes in those with CV catheters, CV-ports, and PICC were *Staphylococcus epidermidis* (n=9; 19%), *Staphylococcus aureus* (n=6; 38%), and *S. epidermidis* (n=8; 80%), respectively. Among those with BSI who did not use CVADs, *Escherichia coli* (n=31; 31%) was the most common pathogen, followed by *S. aureus* (n=13; 13%).

Conclusion

Patients with CLABSI showed lower WBC counts and CRP levels than those with BSI who did not use CVADs. *Staphylococcus epidermidis* was among the most common microbes in CLABSI and accounted for the majority of yielded microbes in patients who used PICC.

## Introduction

The blood culture (BC) test is a first-line diagnostic examination for detecting bacteremia and fungemia in patients with suspected bloodstream infections (BSI) [1]. The annual number of episodes of BSI in Europe is estimated to be over 1,000,000 cases, and over 500,000 cases in the USA [2]. Approximately 10% to 20% of the cases of BSI result in death [3,4], and BSI is one of the most common causes of death in advanced countries. Meanwhile, the timing and ways of performing BC testing differ between countries and clinicians, and further optimizations for the performance of BC tests are desired [5-7]. Correct approaches in sampling and interpreting BC testing to achieve higher sensitivity and a lower risk of contamination are essential for optimizing diagnosis and treatment [8]. One of the difficult situations in making the diagnosis of BSI is when the patients are using central venous access devices (CVADs). Patients with central line-associated bloodstream infection (CLABSI) are known to present with different microbe profiles on BC testing from those without CVADs [9,10]. However, the yielded microbes largely depend on the clinical background of the patients, and the BC result profiles in CLABSI still remain elusive. Therefore, this study evaluated the BC test results of patients with BSI who were treated at a large university hospital in Japan. The data were stratified by usage and types of CVADs to clarify the laboratory features and bacteriological profiles in CLABSI.

## Materials and methods

Participants and evaluated variables

**Figure 1:**
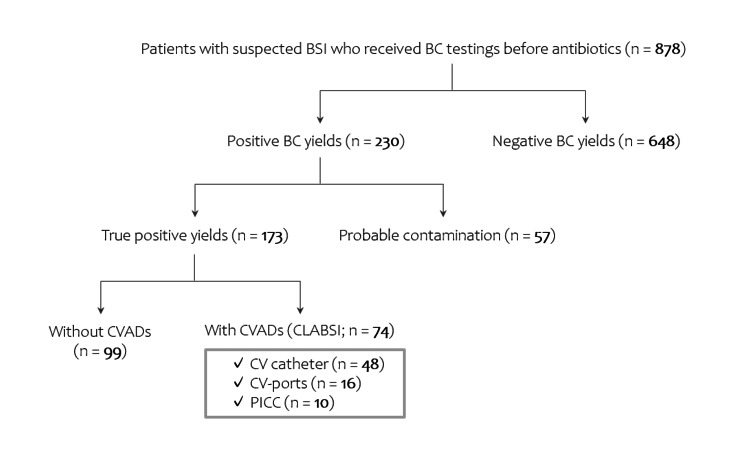
Flow diagram of the study design Patients who were clinically suspected for BSI and tested with BC during the study period were enrolled. They were divided into those with positive and negative BC yields. Those with positive BC yields were further divided into those with definitely true yields and possible contaminants. The presence and types of CVADs were investigated among those with true positive BC yields. BC: Blood culture; BSI: Bloodstream infection; CVADs: Central venous access devices; CV: Central venous; PICC: Peripherally inserted central catheter

Participants in this study were patients of all ages who were clinically suspected of BSI and underwent BC testing before starting treatment with antibiotics at the Tohoku University Hospital, Japan, between April 2020 and September 2020. Demographic data (age, sex), blood test data from BC testing, positivity of BC results, and details of the BC yields were collected from these patients. Systemic inflammation markers (total WBC count and serum C-reactive protein (CRP)) levels) were evaluated from the blood tests. For those with prolonged or recurrent BSI, data at the diagnosis of BSI were used. For patients with positive BC yields, additional clinical information, including the use of CVADs, was further collected. The CVADs were categorized into three types: central venous (CV) catheter, CV access port, and peripherally inserted central catheter (PICC). A flow diagram of the study design is shown in Figure 1.

Culture contamination

Contamination was suspected when only one of the obtained blood samples yielded microbes. Especially when the cultured microbes are coagulase-negative staphylococci, corynebacteria, micrococci, or α-hemolytic streptococci, isolations of microbes from ≥2 blood samples obtained from separate venipunctures were required to classify as true BSI [11]. Yields of other microbes that are rare contaminants, such as gram-negative bacteria or fungi, were assessed on a case-by-case basis by the hospital infection control team when they grew from only one BC set. When the patients had CVADs, a diagnosis of CLABSI was given when the same organism grew from both peripheral vein and catheter blood samples [12]. Patients with the same organism growing from a peripheral vein and catheter tip were also diagnosed with CLABSI.

Statistical analysis

Quantitative data between two independent groups were compared using the Mann-Whitney U test. A comparison of the frequency between two independent groups was performed using the chi-square test or Fisher’s exact test, according to the sample size in each cell. In the multiple linear regression analyses, either the serum CRP level or WBC count was used as the outcome variable, whereas age, sex, true yields of gram-positive organisms, use of a CV catheter, use of a PICC, use of a CV port, and administration of immunosuppressants were used as the explanatory variables. Statistical analyses were performed using R Statistical Software version 4.0.5 (R Foundation, Vienna, Austria). Statistical significance was set at p<0.05.

Ethics

This study was approved by the Institutional Review Board of the Tohoku University Graduate School of Medicine, Japan (approval no. 2020-4100). All study procedures were performed in accordance with the current version of the Declaration of Helsinki. Written informed consent was waived by the Institutional Review Board, and informed consent was obtained in an opt-out manner.

## Results

Participants

Data from 878 patients who were clinically suspected for BSI and underwent BC testing before starting antibiotics during the study period were initially collected. Among them, 230 had positive BC results from at least one set of BC bottles, and the remaining 648 had negative yields. In the 230 positive yields, 173 (75%) were judged to be with clinically-relevant true positive pathogens, and 57 (25%) were judged to be with possible contamination. The demographic and laboratory features of the 173 patients with BSI and 648 patients with negative BC yield are summarized in Table 1. All evaluated variables, including the WBC count (effect size r = 0.06; p = 0.0882) and CRP level (r = 0.04; p = 0.2753), did not significantly differ between those with and without BSI.

**Table 1:** Demographic and laboratory data between patients with and without BSI * Median and interquartile range (25% to 75%) † Mann-Whitney U test. Other comparisons were performed using the chi-square test. BC: Blood culture; BSI: Bloodstream infection; CRP: C-reactive protein

Characteristics	Patients with BSI (n = 173)	Negative BC testing (n = 648)	P-value
Male, n (%)	103 (60%)	359 (55%)	0.3745
Age *	62 (49-73) years	61 (41-72) years	0.0843^†^
Serum CRP levels (mg/dL) *	5.9 (2.0-12.1)	5.3 (1.2-11.6)	0.2753^†^
WBC count (/μL) *	7400 (4700-12,100)	8600 (5200-13,100)	0.0882^†^
WBC ≥11,000 /μL, n (%)	51 (29%)	228 (35%)	0.1878
WBC with 4000-11,000 /μL, n (%)	86 (50%)	297 (46%)	0.4108
WBC <4000 /μL, n (%)	36 (21%)	123 (19%)	0.6656

Bloodstream infection with and without CVADs

Next, the demographic and laboratory data between patients with BSI who used CVADs (CLABSI; n = 74) and those who did not use CVADs (other BSI; n = 99) at the BC testing were compared (Table 2). Patients with CLABSI were younger (effect size r = 0.23; p = 0.0023, Mann-Whitney U test), with lower serum CRP levels (r = 0.23; p = 0.0024), and lower WBC count (r = 0.20; p = 0.0082).

**Table 2:** Demographic and laboratory data between CVAD-related and non-CVAD-related BSI * Median and interquartile range (25% to 75%) † Including chemotherapy in patients with malignancies ‡ Mann-Whitney U test. Other comparisons were performed using the chi-square test. BC: Blood culture; BSI: Bloodstream infection; CRP: C-reactive protein

Characteristics	CLABSI (n=74)	Other BSI (n=99)	P-value
Male, n (%)	50 (68%)	53 (54%)	0.0884
Age *	56.5 (42-71) years	66 (53-77) years	0.0023^‡^
Use of immunosuppressants, n (%)^ †^	25 (34%)	24 (24%)	0.2272
Serum CRP levels (mg/dL) *	3.7 (0.79-7.5)	6.9 (2.3-14.0)	0.0024^‡^
WBC count (/μL) *	6200 (3800-9900)	9200 (5050-13,050)	0.0082^‡^
WBC ≥11,000 /μL, n (%)	16 (22%)	35 (35%)	0.0732
WBC with 4000-11,000 /μL, n (%)	39 (53%)	47 (47%)	0.5984
WBC <4000 /μL, n (%)	19 (26%)	17 (17%)	0.2404

Multivariate analyses

Based on the findings with the univariate analyses that serum CRP level and WBC count were lower among the patients with BSI who used CVADs compared to those who did not use them, multivariate analyses were further performed by using the age, sex, gram-positive BC yields (BC-GP), use of CVADs, and use of immunosuppressants as the explanatory variables (Table 3). The multivariate analyses confirmed that the use of CVADs was associated with lower WBC count (β = -0.202; p = 0.0148) and CRP level (β = -0.184; p = 0.0232), whilst the BC-GP was not.

**Table 3:** Multiple linear regression analysis for WBC count and serum CRP in BSI * p<0.05; ** p<0.01 BC-GP: Gram-positive blood culture; CRP: C-reactive protein; CVADs: Central venous access devices; VIF: Variance inflation factor

Characteristics	Std β	t-score	P- value	VIF
Outcome variable: WBC count				
(Intercept)	0	3.74	0.0003 **	NA
Age	0.095	1.22	0.2230	1.078
Male	–0.028	–0.37	0.7115	1.042
BC-GP	0.114	1.44	0.1505	1.112
CVADs	–0.202	–2.46	0.0148 *	1.188
Immune-suppressants	–0.030	–0.39	0.6948	1.015
Outcome variable: CRP				
(Intercept)	0	1.23	0.2200	NA
Age	0.205	2.68	0.0080 **	1.078
Male	–0.006	–0.08	0.9383	1.042
BC-GP	–0.005	–0.06	0.9515	1.112
CVADs	–0.184	–2.29	0.0232 *	1.188
Immune-suppressants	–0.016	–0.22	0.8276	1.015

Microbe profiles of BSI by the types of CVADs

Lastly, to clarify the background underlying the lower WBC count and CRP level in those with BSI who use PICC, the yielded microbes by the use of CVADs and the types of the used CVADs were further investigated. The list of the identified microbes with BC in the order of the frequencies by the presence and type of CVADs is shown in Table 4. In those with CVADs, *Staphylococcus epidermidis* (n = 19; 26%) was the most common yield, followed by *Staphylococcus aureus* (n = 13; 18%). The rate of *S. epidermidis* was significantly higher in those with PICC (80%) than in those with CV catheters (19%; p = 0.0004, Fisher’s exact test) or CV ports (13%; p = 0.0010). Only two patients (2.0%) of the 99 patients without CVADs were with *S. epidermidis*. For reference, among the excluded 57 individuals with probable BC contamination, the most common contaminant was *S. epidermidis* (n = 20; 35%).

**Table 4:** Microbe profiles of bloodstream infection by the type of central venous access devices The list of identified blood culture yields after excluding probable contaminations is shown by the presence and types of CVADs at the time of BC testing. The identified microbes are listed in the order of frequency. BSI: Bloodstream infection; CLABSI: Central line-associated bloodstream infection; CVAD: Central venous access devices; PICC: Peripherally inserted central catheter, BC: Blood culture

Microbe species	Number (%)
CLABSI total (n = 74)	
1. *Staphylococcus epidermidis*	n = 19 (26%)
2. *Staphylococcus aureus*	n = 13 (18%)
3. *Staphylococcus hominis*	n = 6 (8.1%)
Others	n = 36 (49%)
With CV catheter (n = 48)	
1. *S. epidermidis*	n = 9 (19%)
2. *S. aureus*	n = 7 (15%)
3. *S. hominis*	n = 5 (10%)
4. *Corynebacterium striatum*	n = 4 (8.3%)
Others	n = 23 (48%)
With CV ports (n = 16)	
1. *S. aureus*	n = 6 (38%)
2. *Escherichia coli*	n = 2 (13%)
2. *S. epidermidis*	n = 2 (13%)
Others	n = 6 (38%)
With PICC (n = 10)	
1. *S. epidermidis*	n = 8 (80%)
Others	n = 2 (20%)
BSI without CVADs (n = 99)	
1. *E. coli*	n = 31 (31%)
2. *S. aureus*	n = 13 (13%)
3. *Klebsiella pneumoniae*	n = 4 (4.0%)
3. Group B Streptococcus (*Streptococcus agalactiae*)	n = 4 (4.0%)
3. *Staphylococcus capitis*	n = 4 (4.0%)
Others	n = 43 (43%)

To further clarify the characteristics of BSI with *S. epidermidis*, demographic and laboratory data were compared between those with BSI caused by *S. epidermidis* and those with BSI caused by other microbes. The age was slightly lower in those with *S. epidermidis*, but the difference was not statistically significant (effect size r = 0.15; p = 0.0503, Mann-Whitney U test). The WBC count did not differ between the two groups (r = 0.03; p = 0.7396), but the serum CRP level was lower in those with BSI caused by *S. epidermidis* (r = 0.22; p=0.0054).

## Discussion

The present study demonstrated that S. epidermidis, which is among the most common contaminants of BC testing, was also one of the most common causative microbes in CLABSI, especially in those with PICC. Patients with BSI caused by S. epidermidis showed significantly lower WBC count and CRP level compared to other patients with different causative microbes, which could be the reason why patients with CLABSI who were using PICC showed significantly lower WBC count and CRP level compared to other patients with BSI. These findings imply the importance of not carelessly assuming the BC yield of S. epidermidis as a contaminant, especially in those with CVADs. Furthermore, normal levels in WBC count and CRP level would not guarantee the absence of BSI, especially in those with CVADs. Furthermore, the obtained results indicated the necessity of routinely obtaining at least two sets (four bottles) of blood cultures, especially in those with CVADs because they are likely to have true BC yields of S. epidermidis. A previous study demonstrated that the serum procalcitonin (PCT) level is lower in gram-positive than in gram-negative bacteremia, reflecting a higher inflammatory response by gram-negative microbes [13]. In the study by Koizumi et al. [13], patients with BSI caused by S. epidermidis showed the lowest serum CRP level among the evaluated five major gram-positive and gram-negative microbes, which was consistent with the findings in the present study.

The most commonly used CVAD among the patients with BSI in this study was the CV catheter, followed by the CV port, and PICC was the most uncommon device. However, the present study could not collect data regarding the total number of patients with BSI using each CVAD during the study period; therefore, the risk of developing CLABSI with each type of CVAD could not be determined in this study. A recent study demonstrated that the risk of developing CLABSI was significantly higher with a CV catheter (4.9%) compared to a PICC (2.8%) [14]. An inconsistency between the findings of Pitiriga et al. [14] and the present study was the commonly yielded microbes among the patients with CLABSI caused by PICC; Candida spp. was the most common in the previous study, whereas S. epidermidis was the most common in the present study. There was another previous study evaluating the yielded microbes among patients with CLABSI caused by CV catheters, which revealed that the most common CV catheter-related CLABSI pathogens were S. aureus, Pseudomonas aeruginosa, and non-albicans Candida. These studies, including the present study, with different spectrums of yielded microbes may suggest that the expected pathogens of BSI would largely differ by the background of the participants, including the underlying diseases. However, S. epidermidis is one of the most common microbes seen in patients with implanted prostheses and other indwelling medical devices [15]. Although the BSI caused by S. epidermidisis usually not life-threatening, it is difficult to treat, and both the commensal and infectious lifestyles of this bacterium need to be understood [16]. Putative virulence determinants that may contribute to the colonization and survival of the microorganism include antibiotic-resistant genes and immune evasion molecules [17,18]. A biofilm formation is one of the key elements of immune evasion in BSI caused by S. epidermidis [19,20], which may facilitate the risk of Candida dissemination and resistance to antibiotics [16,21]. Both S. epidermidis and Candida spp. are better remembered as potentially important pathogens underlying CLABSI and closely associated with each other. It was once estimated that more than one billion US dollars were spent annually in the United States alone to treat patients with vascular catheter-related BSI caused by S. epidermidis [16,20]. Moreover, this bacterium is also suggested to play a role as a reservoir for the transfer of genetic elements to enhance pathogenic efficiency in other microbes, including S. aureus [22]. Although S. epidermidis is rare to develop life-threatening BSI, its relatively high frequency and potential role in facilitating BSI caused by other microbes suggest an urgent need to optimize the diagnostic and therapeutic strategies against S. epidermidis in patients with vascular catheter-related BSI, including CLABSI.

Limitations

The present study was performed in a university hospital, and the backgrounds of the enrolled patients and the timing of performing BC testing could differ from those in local and city hospitals. Another limitation is that this study did not evaluate the serum PCT level, which is a more specific biomarker of bacterial infection than WBC count or CRP level. The lack of data on the patients’ underlying diseases and comorbidities or severity is another major limitation of this study. Finally, the overall number of individuals who used CVADs in the university hospital during the study period is unknown; therefore, the risk of developing CLABSI in each of the three types of CVADs remains undetermined.

## Conclusions

Patients with CLABSI showed lower WBC count and CRP level compared to other patients with BSI who did not use CVADs. *Staphylococcus epidermidis*, which was the most common BC contaminant, was also the most common causative microbe in patients with CLABSI. Careful interpretation of BC test yields is required when common contaminants like *S. epidermidis* are obtained from patients who use CVADs.
